# Ovalbumin-sensitized mice have altered airway inflammation to agriculture organic dust

**DOI:** 10.1186/s12931-019-1015-0

**Published:** 2019-03-07

**Authors:** Kristi J. Warren, John D. Dickinson, Amy J. Nelson, Todd A. Wyatt, Debra J. Romberger, Jill A. Poole

**Affiliations:** 1 Pulmonary, Critical Care, Sleep & Allergy Division, Department of Internal Medicine, University of Nebraska Medical Center, 985900 Nebraska Medical Center, Omaha, NE 68198-5910 USA; 2 Veterans Affairs Nebraska-Western Iowa Health Care System, Omaha, NE 68105 USA; 3 Department of Environmental, Agricultural, and Occupational Health, University of Nebraska Medical Center, Omaha, NE 68198 USA

**Keywords:** Asthma, Lung, Inflammation, Agriculture, Airway disease

## Abstract

Agriculture exposures are associated with reducing the risk of allergy and asthma in early life; yet, repeated exposures later in life are associated with chronic bronchitis and obstructive pulmonary diseases. The objective of this study was to investigate the airway inflammatory response to organic dust extract (ODE) in mice with established ovalbumin (OVA)-induced experimental asthma. C57BL/6 mice were either OVA sensitized/aerosol-exposed or saline (Sal) sensitized/aerosol-challenged. Both groups were then subsequently challenged once with intranasal saline or swine confinement ODE to obtain 4 treatment groups of Sal-Sal, Sal-ODE, OVA-Sal, and OVA-ODE. Airway hyper-responsiveness (AHR) to methacholine, bronchiolar lavage fluid, lung tissues, and serum were collected. Intranasal inhalation of ODE in OVA-treated (asthmatic) mice (OVA-ODE) increased AHR and total cellular influx marked by elevated neutrophil and eosinophil counts. Flow cytometry analysis further demonstrated that populations of CD11c^hi^ dendritic cells (DC), CD3^+^ T cells, CD19^+^ B cells, and NKp46^+^ group 3 innate lymphoid cells (ILC3) were increased in lavage fluid of OVA-ODE mice as compared to ODE or OVA alone. Alveolar macrophages, DC, and T cells were significantly increased with co-exposure to OVA-ODE as compared to OVA alone. Lung ILC2 and ILC3 were only increased in OVA-Sal mice. Cytokine/chemokine levels varied with exposure to OVA-ODE reflecting an additive mixture of the pro- and allergic-inflammatory profiles. Collectively, ODE increased airway inflammatory cells and chemotactic mediator release in allergic (OVA) sensitized mice to suggest that persons with allergy/asthma be identified and warned prior to the occupational exposure of potentially worsening airway disease.

**Electronic supplementary material:**

The online version of this article (10.1186/s12931-019-1015-0) contains supplementary material, which is available to authorized users.

## Background

Underlying chronic airway inflammatory diseases, such as chronic bronchitis, asthma and obstructive pulmonary disease, are common among rural agricultural workers [1, 2]. In the United States, large, confined animal feeding operations produce complex bioaerosols composed of gram positive and gram negative bacteria, fungal spores, and particulates capable of eliciting pro-inflammatory respiratory responses [3–6]. Several studies define the airway inflammatory response following acute and repetitive complex swine confinement organic dust extracts (ODE) in animal models. These studies demonstrate neutrophil, macrophage, and lymphocyte influx, airway hyper-responsiveness (AHR), and release of pro-inflammatory cytokines, including tumor necrosis factor (TNF)-α, interleukin (IL)-6, and neutrophil chemoattractants (CXCL1 and CXCL2), which resemble the human disease [7–10].

It is known that early life farming exposures can reduce the development of allergic asthma in children in farming communities [11, 12]. It is also shown that mice instilled with household dust extracts from the homes of rural Amish farmers, prior to and during experimental ovalbumin (OVA) allergic sensitization and challenge, demonstrated reduction in AHR and eosinophilia [13], supportive of the hygiene hypothesis. In contrast, exposures to larger, industrial farming environments (e.g. swine confinements containing over 500 animals per facility) are associated with non-allergic asthma symptoms and self-reported asthma as high as 50% in children [14]. The actual prevalence of asthma among adults engaged in farm work is variable, ranging from 5 to 13% [15, 16]. The “healthy worker effect” has been ascribed the farming industry as it is thought that farmers with poor respiratory health leave the industry whereas those without respiratory health disease remain. In support of this effect, a longitudinal study by Thaon and colleagues [17] demonstrated that *former* farmers had increased doctor-diagnosed asthma (OR 7.51; CI 1.59–35.41) as compared to administrators, whereas *current* farmers had similar risk of doctor-diagnosed asthma (OR 0.93; CI; CI:0.15–5.81) as compared to administrators. Less is known about the impact of agriculture exposures on those with pre-existing asthma. It has been shown that among farm operators with farm work-related asthma, 33% had asthmatic exacerbations while doing farm work, suggesting that farm exposure is a risk factor for worsening asthma disease [16]. Thus, studies examining the complexities of immune pathogenesis of complex industrial animal farming exposures in allergic airway diseases might be important for preventative and/or therapeutic strategies in identified, at-risk exposed persons.

Animal studies are useful tools to investigate airway inflammatory responses, and in particular, the murine ovalbumin (OVA)-sensitization and challenge protocol is utilized routinely to study experimental allergic asthma. Whereas the organic dust airway inflammatory model resembles a mix of T helper 1 (Th1) and Th17 cell mediated airway inflammatory responses [9, 18], allergic asthma is characterized by a type 2 response involving eosinophils, type 2 helper T (Th2) cells, group 2 innate lymphoid cell (ILC2) influx, with release of type 2 cytokines including IL-4, IL-5, and IL-13 [19–21]. In this study, we hypothesized that ODE would worsen allergic airway inflammatory consequences including AHR, airway cellular influx, and release of type 2 mediators established after OVA sensitization and challenge. To test our hypothesis, C57BL/6 mice were sensitized and challenged with OVA according to a 7-day aerosolized ovalbumin protocol to establish allergic inflammation. This was followed 1 day later by a single inhalation exposure to ODE. We found that ODE treatment potentiated several allergic airway inflammatory indices.

## Methods

### Organic dust extract (ODE)

Aqueous organic dust extract (ODE) was collected and prepared as previously described [22]. Briefly, settled surface dust samples (~ 3 ft off ground) from swine confinement animal feeding operations (~ 500–700 animals) were collected and 1 g was placed into sterile Hank’s Balanced Salt Solution (10 ml; Sigma, St. Louis, MO) [22]. Solution was incubated for one hour at room temperature, centrifuged for 20 min at 2000 x *g*, and the final supernate was filter sterilized (0.22 μm), a process that also removes coarse particles. Stock (100%) ODE aliquots frozen at − 20 °C until use in experiments and diluted in sterile phosphate buffered saline (PBS; pH: 7.4; diluent) to a final concentration of 12.5% (vol/vol). The 12.5% ODE concentration in 50 μl volume has been previously shown to elicit optimal lung inflammation in mice and is well-tolerated [7]. These diluted extracts contained approximately 4 mg/ml of total protein as measured by nanodrop spectrophotometry (NanoDrop, Thermo Fisher, Waltham, MA). The endotoxin concentration of this 12.5% ODE ranged from 155 to 175 EU/ml as determined by the limulus amebocyte lysate assay according to manufacturer’s instructions (Sigma). For reference, a recent study of dairy barns in eastern Colorado found that the geometric mean for endotoxin exposure was 438 EU/m^3^, with 89% of workers exceeding the recommended adjusted occupational exposure level of 67 EU/m^3^ [23]. There are no commercially available kits to quantitate gram positive bacterial peptidoglycan levels, but prior shotgun metagenomics analyses of DNA pyrosequencing of dust samples collected from swine facilities revealed a strong presence of gram positive bacteria [3].

### Animal model

**Fig. 1:**
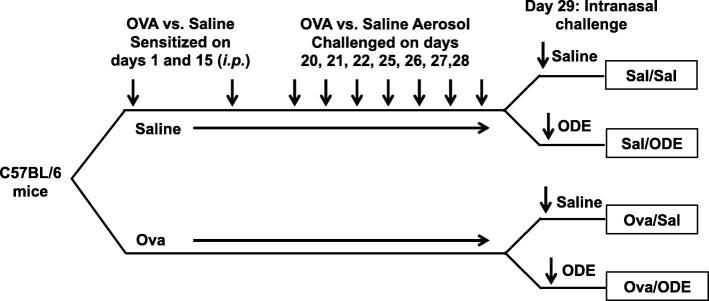
Experimental protocol. C57BL/6 mice were sensitized to ovalbumin (OVA) intraperitoneal (i.p.) on days 1 and 15 and OVA aerosolized challenged on days 20–28. On day 29, mice were intranasally (i.n.) challenged with saline or 12.5% organic dust extract (ODE)

Male C57BL/6 mice (6–8 wk. old) were purchased from The Jackson Laboratory (Bar Harbor, ME) for consistency with prior agriculture exposure animal modeling studies conducted by us [7, 22, 24] and others. [8–10] All animal procedures were approved by the Institutional Animal Care and Use Committee at the University of Nebraska Medical Center and were in accordance with the NIH guidelines for the use of rodents. Mice were sensitized with 100 μl of chicken egg ovalbumin (Grade V; 500 μg/mL) absorbed with aluminum hydroxide (Sigma, 29 mg/mL) (OVA) intraperitoneal (i.p.) on days 1 and 5, and then challenged with aerosolized 1% OVA on days 20, 21, 22, 25, 26, 27 and 28 for 20 min each day. Saline control animals were injected with saline and saline aerosolized. Next, mice were intranasally (i.n.) treated with 12.5% ODE or saline 4–5 h prior to being sacrificed on day 29 (Fig. 1). Mice were lightly anesthetized by isoflurane inhalation before intranasal inhalation of sterile saline (PBS) or 12.5% ODE per previously established procedure [7].

### Pulmonary function measurement

Based upon previous work determining optimal time point to determine ODE-induced inflammatory responses [7], 3 h following the one-time treatment with intranasal inhalation of 12.5% ODE or saline, mice were anesthetized, tracheostomized, and mechanically ventilated at a rate of 160 breaths/min, and tidal volume of 0.15 ml, using a computerized small animal ventilator (Buxco Finepointe; DSI, St. Paul, MN). Dose-responsiveness to aerosolized methacholine (1.5–24.0 mg/ml) was obtained and results reported as total lung resistance (R_L_).

### Bronchoalveolar lavage and Cytospin

In separate studies, bronchoalveolar lavage (BAL) fluid was collected using 3 × 1 ml PBS. Total cell numbers from pooled lavages were enumerated by hemacytometer and differential cell counts were determined on cytospin-prepared slides (Cytopro Cytocentrifuge, ELITechGroup, Logan, UT) stained with DiffQuick (Thermo Fisher).

### Cytokine/chemokine detection

Cell-free BAL fluid was evaluated for cytokines and chemokines by ELISA. Levels of mediators previously implicated in mediating ODE-induced airway inflammation include TNF-α, IL-6, and the murine neutrophil chemoattractants, CXCL1 and CXCL2, [15] were quantitated by Quantikine ELISAs from R&D Systems (Minneapolis, MN) according to manufacturer’s instructions. Levels of cytokines classically associated with allergic disease including IL-4, IL-5, and IL-13 were quantitated using the Ready-SET-go ELISA kits (Affymetrix, Thermo Fisher). Levels of chemokines including CCL2, CCL3, CCL8, CCL11, CCL12, and CCL22, and levels of cytokines including IL-9, IL-17A, IL-17E, and IL-33, were quantitated by murine Luminex multiplex assay from R&D Systems according to manufacturer’s instructions.

### Serum IgE

Whole blood was collected at time of euthanization from axillary artery and placed in BD Microtainer Tubes (Becton Dickinson, Franklin Lakes, NJ), centrifuged and cell-free serum collected. Serum IgE levels were quantified by ELISA kit (Mouse IgE ELISA Set, BD Biosciences, San Diego, CA) according to the manufacturer’s instructions. The serum from the saline treated/saline challenged mice was diluted 1:2, and serum from the OVA-treated/ODE-challenged mice was diluted 1:20.

### Histopathology

Following lung lavage, whole lungs were excised and inflated to 15 cm H_2_O pressure with 10% formalin (Sigma) to preserve pulmonary architecture. Lungs were embedded in paraffin and sections (4–5 μm) were cut and stained with hematoxylin and eosin (H&E). To determine mucus in airway epithelial cells, Muc5ac intracellular levels were detected using UEA1-lectin conjugated with fluorophore 555 nm in separate unstained slides, and nuclei were counterstained with DNA with 4′,6 diamidino-2-phenylindole (DAPI) as previously described [25]. Intracellular mucin was assessed by volumetric density analysis using ImageJ software and quantified from images of multiple fields at 20X magnification as previously described [26].

### Cell staining and flow cytometry

In separate studies, BAL fluid and whole lung tissues were separately collected and processed for cellular identification by flow cytometry. Whole lung tissues were harvested after the right ventricle was infused with 10 mL of sterile PBS with heparin (1.5 units/ml; Sigma) to remove blood from the pulmonary vasculature. Lung tissues were subjected to an automated dissociation procedure using the gentleMACS tissue dissociator (Miltenyi Biotech, Auburn, CA) as previously described [22]. After passing cell solution through nylon mesh (40 μM; Fisher) and red blood lysis using a 0.84% (*w*/*v*) ammonium chloride treatment (5 min at 4 °C), cells were resuspended in PBS, and lung cells were isolated by density gradient centrifugation over Ficoll-Paque PLUS (GE Healthcare, Chicago, IL). Viability of the lung cells was assessed by trypan blue exclusion and LIVE/DEAD fixable Violet Dead Cell Stain Kit (Life Technologies, Thermo Fisher). Ultimately, less than 1% of gated cells were not viable, with no difference in viability noted between treatment conditions (data not shown). Total lung cells for each animal were enumerated by the TC20 automated cell counter (Bio-Rad, Hercules, CA).

Bronchoalveolar lavage fluid (BALF) and lung cells from each animal were incubated with CD16/32 (Fc Block, BD Biosciences) to minimize non-specific antibody staining then stained with monoclonal antibodies (mAb) directed against murine CD45 (clone 30-F11), CD3e (clone: 145-2C11), CD19 (clone: ID3), CD11b (clone: M1/70), CD11c (clone: N418), Ly6G (clone 1A8), Siglec^−^F^+^ (clone E50–2440), NK1.1 (clone: PK136), ST2 (clone: RMST2–2), NKp46 (clone 29A1.4), and ICOS (clone: 7E.17G9). CD45, CD3e, CD11b, Siglec F^+^, and ICOS antibodies obtained from BD Biosciences. CD11c, NK1.1, ST2, and CD19 antibody obtained from Affymetrix. Ly6G obtained from BD Pharmingen, and NKp46 obtained from Biolegend (San Diego, CA). Compensation was performed with antibody capture beads (BD Biosciences) stained separately with each individual mAb used in test samples. Cellular data was acquired on the BD Fortessa (BD Biosciences) and subsequently analyzed using Flow Jo software version 9 (Ashland, OR).

All populations were gated by forward- and side-scatter properties characteristic of lymphocytes (SSC-FSC^+^) and granulocytes (SSC^+^FSC^lo^) and antibody-specific staining fluorescence intensity. The gating strategy after removal of debris, doublets, and selection of CD45^+^ cells was CD3^+^CD19^−^ T cells, CD3^−^CD19^+^ B cells, and CD3^−^CD19^−^CD11c^hi^ conventional DC, SSC^+^Autoflourescence^+^CD11c^+^ macrophages, and CD3^−^NK1.1^+^ natural killer cells (NK cells), CD11c^−^CD11b^+^Siglec^−^F^+^ eosinophils, CD11c^−^Ly6G^+^ neutrophils as previously published [27, 28]. Group 2 innate lymphoid cells (ILC2) were gated as lineage (LIN) negative (CD3^−^CD19^−^CD11b^−^CD11c^−^NK1.1^−^) and inclusion of ICOS^+^ST2^+^, and ILC3 were gated as LIN^−^NKp46^+^. Gating strategy for leukocytes are shown in Additional file 1: Figure S1. The percentage of all respective cell populations were determined from CD45^+^ lung leukocytes, and this percentage was multiplied by the respective total cells acquired from each BALF or whole lung to determine specific cell population numbers for each animal.

### Statistical Methods

Data are presented as the mean ± standard error where indicated. To detect significant changes between two groups, statistics were performed using Students t-test or two-tailed, Mann-Whitney test, as appropriate. For experiments with 3 or more groups, one-way analysis of variance (ANOVA) with post-hoc tests (Tukey) was done for multi-comparison within the groups. To analyze the methacholine dose response curves, we used a two-way ANOVA (because there are 2 independent variables: treatment group and dose of methacholine) followed by Mann-Whitney nonparametric test when group differences were significant, *P* < 0.05. Statistical analysis was performed using GraphPad Prism software (La Jolla, CA) and/or SPSS software (SPSS, Chicago, IL) and significance was set at *P* < 0.05.

## Results

### Airway hyper-responsiveness in mice co-exposed to ova-allergen and ODE

**Fig. 2:**
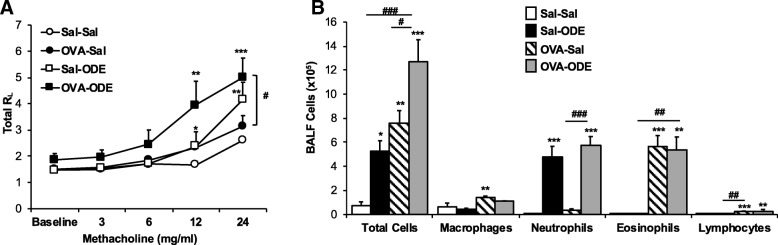
Airway hyper-responsiveness and inflammatory response with co-exposure of OVA-induced asthma and ODE. **a**, Three hours following i.n. ODE or saline challenge, airway hyper-responsiveness (AHR) to aerosolized methacholine (0/baseline, 3, 6, 12, 24 mg) was measured and line graph represents mean (± SEM) total lung resistance (R_L_). **b**, At 4–5 h post ODE or saline i.n. exposure, cellular influx into the bronchoalveolar lavage fluid (BALF) was determined. Bar graph represents mean with standard error bars shown. *N* = 7–8 mice/group from 2 independent experiments. Statistical significance denoted (*P < 0.05, **P < 0.01, ***P < 0.001) vs. Sal-Sal. Statistical significance denoted (# *P* < 0.05, ## *P* < 0.01, ### *P* < 0.001) as indicated

Consistent with previous work [7], ODE exposure alone (Sal-ODE) induced an increase in AHR to methacholine as compared to Sal-Sal control (Fig. 2a). Mice sensitized and challenged with OVA (OVA-Sal) did not demonstrate a significant increase in AHR as compared to Sal/Sal animals. This lack of AHR in C57BL/6 mice OVA sensitized and challenged has been observed by others [29, 30]. However, with co-exposure (OVA-ODE) there were significant increases in AHR at methacholine doses 12 mg/ml (*P* < 0.05) and 24 mg/ml (*P* < 0.001) as compared to Sal-Sal control mice. There was also an increase in AHR in OVA-ODE animals as compared to OVA-Sal at 12 and 24 mg/ml methacholine (*P* < 0.05), suggesting that ODE exposure activates AHR in OVA-treated mice.

### Airway inflammatory cell influx with OVA-allergen and ODE

There were significant increases in total cell influx following single exposure to ODE (Sal-ODE), OVA-treatment (OVA-Sal), and co-exposure (OVA-ODE) as compared to saline control (Sal-Sal) (*P* < 0.05; Fig. 2b). Neutrophil influx, but not eosinophilic influx, was increased in Sal-ODE treatment groups compared to the Sal-Sal (*P* < 0.001), which is a characteristic feature of ODE-induced airway inflammation [31]. Macrophages, eosinophils, lymphocytes, but not neutrophils, were significantly increased in the OVA-Sal treatment group in comparison to Sal-Sal group, consistent with experimental allergic asthma in C57BL/6 mice [29, 30]. Airway total cellular influx was significantly increased (*P* < 0.05) in the OVA-ODE co-exposure treatment group as compared to all other treatment groups, and marked by increased numbers of macrophages, eosinophils, neutrophils, and lymphocytes.

### Serum IgE levels and lung histopathology with OVA and ODE

**Fig. 3:**
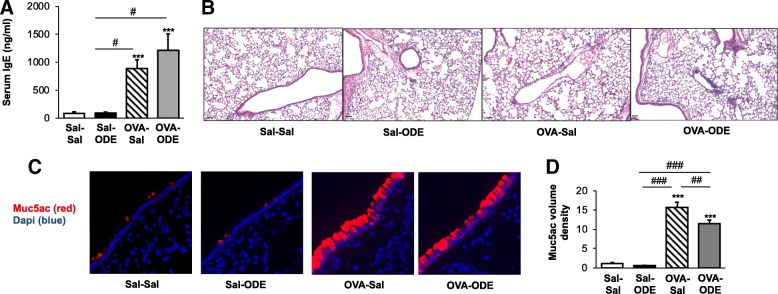
Serum IgE levels and lung histopathology from OVA and ODE treated animals. **a**, Murine serum IgE levels from mice from each treatment group were determined with bar graph represents mean with standard error bars shown. *N* = 7–8 mice/group from 2 independent experiments. **b**, A representative murine lung section (hematoxylin and eosin stain, 10X magnification) from each treatment group is shown. Line scale is 60 μm. Note that there is peribronchiolar and perivascular cellular cuffing in OVA and ODE treatment groups. **c**, Representative images from immunostaining of lung tissues for Muc5ac (UEA1-lectin). Note increased mucin staining in OVA treated groups. **d**, Bar graph represents mean with standard error bars of Muc5ac volume density as determined by ImageJ software analysis (*n* = 28–31 images/group from 2 independent experiments). Statistical significance denoted (***p < 0.001) vs. Sal-Sal. Statistical significance denoted (#*p* < 0.05, ##*p* < 0.01, ###*p* < 0.001) as indicate

OVA sensitization and challenge (OVA-Sal) resulted in an increase in serum IgE levels, consistent with experimental allergic asthma (Fig. 3a). Mice subsequently challenged with ODE (OVA-ODE), also demonstrated increased serum IgE levels, and there was no difference between OVA-Sal and OVA-ODE. There was also no difference in IgE levels in Sal-ODE versus Sal-Sal treatment animals. By microscopic review, there was evidence of perivascular and peribronchiolar cuffing with infiltration of cells into the lung parenchyma in Sal-ODE, OVA-Sal, and OVA-ODE treatment groups as compared to Sal-Sa (Fig. 3b). The gel-forming mucin, Muc5ac, is overproduced and secreted by airway epithelial cells in human allergy and allergic mouse models [32]. There was a striking increase in Muc5ac intracellular staining in OVA-Sal and OVA-ODE, but not Sal-ODE, treated mice as compared to Sal-Sal treated animals (Fig. 3c). These observations were confirmed with volume density quantification by ImageJ software analysis (Fig. 3d), and interestingly, there was a slight, but significant decrease in Muc5ac staining in OVA-ODE as compared to OVA-Sal.

### Impact of ODE challenge on the recruitment of DC and lymphoid cells in the lavage fluid and lungs of OVA-challenged mice

**Fig. 4:**
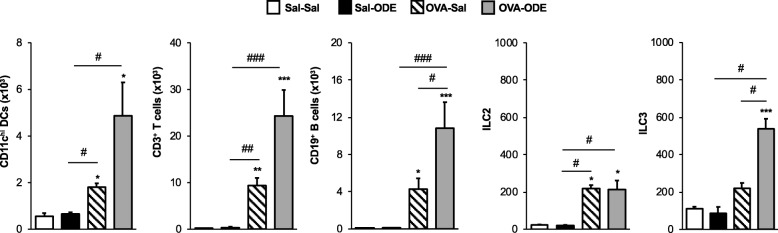
Dendritic cell and lymphoid cell airway influx induced by OVA and ODE exposures. BAL fluid from each treatment group was processed and analyzed by flow cytometry. Numbers of cells were calculated by multiplying the percentage of cells in respective gate (% of CD45 ^+^cells as analyzed by FACS) multiplied by respective total cells for each mouse. Bar graphs depict means with standard error bars of CD11c^hi^ DC, CD3^+^ T cells, CD19^+^ B cells, ILC2 (LIN^−^ICOS^+^ST2^+^), and ILC3 (LIN^−^NKp46^+^). *N* = 4–6 mice/group. Statistical significance denoted (* *P* < 0.05, ** *P* < 0.01, *** *P* < 0.001) vs. Sal-Sal. Statistical significance denoted (# *P* < 0.05, ## *P* < 0.01, ### *P* < 0.001) as indicated

To further understand the distribution of inflammatory cells, particularly lymphoid cells, recruited to the airways and lungs of mice, separate studies were conducted to phenotype the airway cellular influx by flow cytometry (see Methods section and Additional file 1: Figure S1). The BAL fluid studies were focused on CD11c^hi^ dendritic cells (DC), total CD3^+^ T cells, CD19^+^ B cells and innate lymphoid cell populations. The OVA allergic mice (OVA-Sal) demonstrated significant increases in DC, T cells, B cells, and ILC2 as compared to Sal-Sal control mice (Fig. 4, *P* < 0.05). There was also a significant increase (*P* < 0.05) in the OVA-ODE treatment group as compared to Sal-Sal for DC, T cells, B cells, ILC2, and ILC3. Whereas ODE alone did not impact these cellular populations, there was increased cellular recruitment following ODE exposure in OVA-challenged mice (OVA-ODE), achieving statistical significance for DCs, T cells, B cells and ILC3 in OVA-ODE vs. OVA-Sal groups (*P* < 0.05). Few NK cells were found in the BAL fluid from any treatment groups, and there was no significant difference among groups (data not shown).

**Fig. 5:**
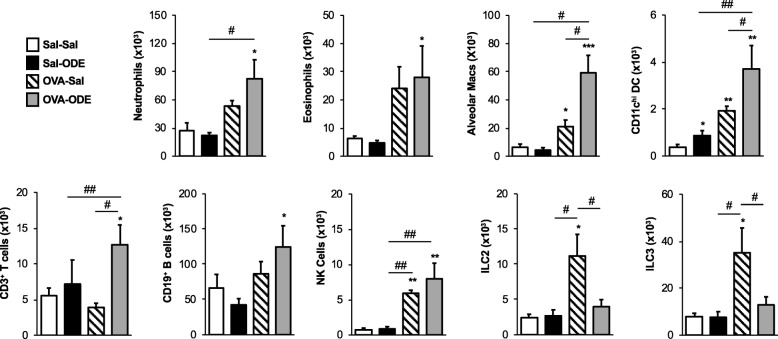
Lung tissue infiltrates induced by OVA and ODE exposures. Whole lung tissues from each treatment group were processed and analyzed by flow cytometry. Numbers of cells were calculated by multiplying the percentage of cells in respective gate (% of CD45 ^+^cells as analyzed by FACS) multiplied by respective total cells for each mouse. Bar graphs depict means with standard error bars of neutrophils (CD11c^−^Ly6G^+^), eosinophils (CD11c^−^CD11b^+^Siglec^−^F^+^), alveolar macrophages (autoflourescence^+^CD11c^+^), conventional CD11c^hi^ DC, CD3^+^ T cells, CD19^+^ B cells, natural killer (NK) cells (CD3^−^NK1.1^+^), ILC2 (LIN^−^ICOS^+^ST2^+^), and ILC3 (LIN^−^NKp46^+^). *N* = 4–6 mice/group. Statistical significance denoted (* *P* < 0.05, ** *P* < 0.01, *** *P* < 0.001) vs. Sal-Sal. Statistical significance denoted (# *P* < 0.05, ## *P* < 0.01) as indicated

Next, whole lung tissue immune cells from mice were analyzed by flow cytometry (Fig. 5). Significant lung neutrophil infiltration was only noted with co-exposure (OVA-ODE), and not demonstrated with ODE alone (Sal-ODE), suggesting that OVA sensitization/challenged mice were primed for an enhanced ODE-induced lung neutrophil response. Whereas eosinophils were significantly increased in the BAL fluid of OVA-Sal treated mice (Fig. 4), eosinophilic infiltration into the lung tissue was not significantly increased (*P* = 0.1). Lung eosinophil levels were significantly increased in the OVA-ODE treatment group (*P* < 0.05) as compared to Sal-Sal mice. Alveolar macrophages were increased in OVA-Sal mice (*P* < 0.05), and this response was significantly potentiated with co-exposure. Correspondingly, conventional CD11c^hi^ DC were increased in all treatment groups including Sal-ODE, OVA-Sal, and OVA-ODE as compared to Sal-Sal control mice. Additionally, this response was significantly increased (*P* < 0.05) in the OVA-ODE treatment group as compared to OVA or ODE alone. Only the co-exposure (OVA-ODE) treatment group demonstrated significant (*P* < 0.05) increases in lung CD3^+^ T cells and CD19^+^ B cells. Lung innate lymphoid cells also demonstrated differences among treatment groups. NK cells were increased in both OVA-Sal and OVA-ODE, but increases in ILC2 and ILC3 populations were only found in the OVA-Sal treated mice. Collectively, these support a compartmentalized airway versus interstitial inflammatory response between BAL fluid and whole lung tissue.

### Cytokine and chemokine expression varies by independent treatment with OVA, ODE and with co-exposure to ODE and OVA

Because significant differences in airway cellular populations were demonstrated in the murine treatment groups, chemokines implicated in recruitment of various cellular populations and specific cytokines produced by these various populations was investigated [19, 21]. We hypothesized that we would detect a unique cytokine/chemokine profile associated with each treatment group, and that the combination of OVA and ODE exposures would exacerbate allergic inflammation in the form of amplified IL-4, IL-5, IL-13, CCL22 and CCL11. Consistent with prior work [7], we confirmed that a one-time ODE inhalant exposure (Sal-ODE) induces TNF-α, IL-6, and the murine neutrophil chemoattractants, CXCL1 and CXCL2 (Table 1). Furthermore, ODE treatment alone (Sal-ODE treatment group) also induced CCL2 and CCL3, which have also been implicated in neutrophil recruitment [19]. OVA alone (OVA-Sal treatment group) induced IL-4, IL-5, IL-13, CCL3, CCL11, CCL12 and CCL22. IL-4, IL-15, and IL-13 are classic cytokines associated with Th2 allergic inflammation, and CCL3, CCL11, CCL12, and CCL22 are important in lymphocyte, macrophage, and eosinophil recruitment following allergen challenge [19]. In the OVA-ODE co-exposure treatment group, TNF-α, IL-6, IL-4, IL-13, CCL2, CCL3, CCL12, CCL22, CXCL1 and CXCL2 were all significantly increased as compared to Sal-Sal treatment groups (*P* < 0.05). Of these induced proteins in the OVA-ODE treatment group, IL-6, IL-4, IL-13, CCL12, and CCL22 were increased above levels demonstrated in the Sal-ODE or OVA-Sal treatment groups, suggesting a synergistic response. Interestingly, there was a reduced TNF-α response in the OVA-ODE treatment group as compared to Sal-ODE, and IL-5 and CCL11 were the only proteins induced by OVA treatment that were not subsequently amplified by ODE treatment. Levels of IL-9, IL-17A, IL-17E, IL-33, and CCL8 were not detected.

**Table 1:** Levels of inflammatory cytokines and chemokines in the BAL fluid of mice from treatment groups

Protein	Sal-Sal	Sal-ODE	OVA-Sal	OVA-ODE
TNF-α	31.6 ± 11.8	471.0 ± 68.9*	28.4 ± 11.5	223.7 ± 36.7*#
IL-6	21.8 ± 6.3	526.6 ± 91.7*	24.0 ± 9.1	1068 ± 120.2*#
IL-4	1.5 ± 0.8	0.8 ± 0.4	3.3 ± 1.0*	8.1 ± 3.2*#
IL-5	1.8 ± 0.6	2.8 ± 1.0	18.5 ± 7.8*#	7.1 ± 2.1
IL-13	0.1 ± 0.1	0.0 ± 0.0	14.6 ± 1.5*#	16.7 ± 2.4*#
CCL2	9.1 ± 0.4	36.1 ± 11.9*	14.5 ± 3.5	35.7 ± 17.2*
CCL3	15.0 ± 10.0	5493 ± 1065*%	766.1 ± 339.7*	4878 ± 1427 *%
CCL11	3.8 ± 0.5	7.0 ± 1.7	21.1 ± 5.9*	14.2 ± 2.6
CCL12	7.5 ± 0.3	10.0 ± 0.6	129.8 ± 55.3*	231.2 ± 50.7*#
CCL22	13.5 ± 2.4	89.7 ± 35.7	295.0 ± 118.8*	851.9 ± 272.3*#
CXCL1	71.5 ± 19.2	1336 ± 320.1* %	144.7 ± 28.7	1037 ± 307.0*
CXCL2	33.5 ± 13.3	287.9 ± 60.3*%	27.2 ± 14.51	197.0 ± 37.5*%

Values (pg/ml) are mean ± standard error of mean. *N* = 4–8 mice/group

Statistical significance versus Sal-Sal denoted as *(*p* < 0.05); versus Sal-ODE denoted as #(*p* < 0.05) and bolded; versus OVA-Sal %(*p* < 0.05). IL-9, IL-17A, IL-17E, IL-33 and CCL8 were not detected

## Discussion

Airway inflammatory diseases are common among agricultural workers [2, 15]. In this study, we investigated how an acute inhalant exposure to an agriculture acquired organic dust extract (ODE) would impact lung inflammatory responses in a murine experimental allergic asthma model. Overall, we demonstrated that ODE exposure amplifies several airway inflammatory outcomes. These included enhancement of AHR and a strong diversity in inflammatory cellular influx in the airspace and lung tissues corresponding to increased and diverse chemoattractant release. Collectively, these studies support that allergic asthma primes the lung microenvironment response toward an exaggerated response following dusty farm exposures, and thus, asthma represents an important risk factor to identify in persons prior to the initiation of working in farming operations.

Using a standard OVA sensitization and challenge experimental allergic asthma model, we demonstrated that ODE potentiated, not inhibited, several airway inflammatory parameters once allergic asthma was established. Overall, there was an admixture of inflammatory eosinophils and Th2 cytokines representative of allergic disease plus neutrophils and Th1 cytokines representative of dust exposure that was likely responsible for the potentiated pathology. As anticipated, airway mucin expression was increased in the allergic animals, but interestingly, there was a significant decrease in the mucin expression following ODE exposure. It is possible that a one-time ODE challenge enhanced the mucus secretion to explain this decrease in intracellular staining for Muc5ac, but further studies would be warranted to fully understand this observation. Although a single exposure to ODE did not impact mucin levels in the airway, it has been demonstrated that repetitive ODE exposure increases Muc5ac expression but that this expression is not at the magnitude seen in experimental allergic asthma models.

AHR in asthma is complex with the central focus on the growth and reactivity of airway smooth muscle, which is presumably stimulated by allergen-induced inflammatory mediators [33]. In non-allergic asthma, neutrophil recruitment [34] and TNF-α [35] can result in AHR, but AHR can also occur independently of neutrophil recruitment or TNF [36]. Heightened Th1 inflammation with interactions between TNF-α and IFN-γ has also been shown to contribute to corticosteroid resistance in airway smooth muscle [37]. We speculate that combination and diversity of inflammatory mediators demonstrated in the co-exposure model contributes to the potentiated AHR. However, future studies could focus on the role of airway smooth muscle cell function in mixed allergic and nonallergic disease triggers focusing on features including contractility, proliferation, and release of extracellular components and mediators that influence bronchoconstriction and bronchodilation to inform future therapeutics.

Albeit at much lower numbers as compared to neutrophils and eosinophils, there was also increased lavage fluid DCs, T cells, B cells, and ILC3 in allergic asthma mice challenged with ODE. These observations in lavage fluid sampling were not entirely consistent with the inflammatory cellular population determined in the lung tissues. OVA mice challenged with ODE did demonstrate the highest numbers of neutrophils, eosinophils, macrophages, T and B lymphocytes among the treatment groups. However, lung tissue ILC2 and ILC3 infiltrates were not increased with co-exposure, and were only elevated in asthmatic mice. NK cells that were essentially absent in the lavage fluid were found in the allergic asthma mice and co-exposed mice, and there was no difference between these groups.

Innate lymphoid cells are increasingly recognized as critical effectors of innate immunity and can play pivotal roles in the initiation, regulation, and resolution of inflammation [38]. ILC2 parallel Th2 lymphocytes with involvement in allergic responses with expression of allergic effector cytokines including IL-4, IL-5, and IL-13 [38]. Thus, the finding that ILC2 were increased in lavage fluids and lung tissue of allergic asthma mice is anticipated, but ILC2 were not enhanced with ODE exposure. In comparison, ILC3 contribute to bacterial and fungal responses and are also implicated in tissue repair after injury [38]. ILC3 were increased in lung tissues but not lavage fluid of asthmatic mice, and strikingly, this observation was modulated following ODE challenge in asthmatic animals (co-exposure) with increased ILC3 population detected in the lavage fluid but not lung tissue. ODE alone did not impact ILC3 recruitment. The enriched and diverse microbial component nature of ODE [3] could explain the ILC3 response, however, ILC3 recruitment was not observed with ODE exposure alone suggesting that mixed or “two-hit” signals are necessary to engage ILC3. It is possible that in the setting of prolonged/chronic allergy plus dust exposure there might be further involvement of ILC3 responses. NK cells conduct cytotoxic activity and pro-inflammatory cytokine release [38]. It is noted that a one-time ODE exposure did not significantly impact the lung tissue cell infiltrates, except DC, and therefore, the potentiation effect cannot be explained as an additive effect of the co-exposure. The difference between lavage fluid and lung tissue infiltrates could be explained by asthma-induced epithelial cell barrier dysfunction. Disruption of epithelial barrier and leakiness in allergic asthma has been attributed to differences in tight junction structures [39]. ILC2 have been described to breakdown bronchial epithelial barrier integrity [40], and thus, the increased presence of ILC2 infiltrating the lung tissues of the allergic asthma mice may be important for the predisposition to further disease following ODE exposure.

Variations in chemokine responsiveness may also account for the alterations in cellular influx. CCL2 (monocyte chemotactic protein), CCL3 (macrophage inflammatory protein-1α), and the murine neutrophil chemoattractants, CXCL1 and CXCL2, were elevated in ODE exposed mice. CCL3, CCL11 (eotaxin), CCL12 (monocyte chemotactic protein 5), and CCL22 were increased in OVA-challenged mice. These chemokines can recruit and prime eosinophils and Th2 allergic responses [19]. In the co-exposure animals, there was an increase in all chemokines reported, except CCL11, which represents the diversity of cellular populations demonstrated. The potentiated, but not statistically significant, response of CCL12 and CCL22 could explain the heightened lung macrophage and DC response observed with co-exposure. It was also noted that trends were observed with cytokines in co-exposed animals in that TNF-α was reduced, not increased, whereas IL-6 and IL-4 were increased. Further studies could consider potentially isolating specific cell populations to determine polarization and/or cell-specific cytokine/chemokine responsiveness, which might be applicable to future strategies aimed at the development of specific monoclonal therapeutic targeted approaches.

There are limitations to this animal study. Whereas OVA was utilized to elicit allergic asthma due to its widespread use in experimental asthma, OVA is not a natural allergen and future studies could consider use of pollens, dust mites, or molds in modeling allergic asthma. Advantages of using the Th1-dominant C57BL/6 mice is that numerous genetic modifications on this strain background are commercially available to explore future mechanisms and this strain is commonly used in agriculture exposure studies. However, the Th2-dominant BALB/c strain could also be utilized, particularly if AHR is the key endpoint as C57BL/6 mice are more resistant to AHR. Next, agriculture organic dust is complex and comprised of a diverse mixture of gram positive and gram negative microbial components, such that there is not one component (e.g. endotoxin) in the dust that has explained the entirety of the airway disease. Namely, roles for Toll-like receptor 2, 4, 9 and the common adaptor proteins MyD88 and TRIF have been implicated with strongest roles for MyD88 and TRIF [8, 41].

## Conclusion

In conclusion, the finding that acute ODE exposure exacerbated lung inflammation in allergic asthmatic mice supports that identifying or screening persons with allergy/asthma prior to occupational exposure in dusty, agriculture environments is warranted. Identification of at-risk persons would allow preemptive workplace warnings or modifications that include strict use of protective respiratory equipment to minimize disease outcomes. Additionally, recognition of the diverse and complex cellular/mediator response in dusty agriculture environments with allergy is necessary to develop strategies aimed at preventative and/or therapeutic approaches.

## Additional file

Additional file 1: **Figure S1.** A representative dot blot of gating strategy. Isolated lung cells were processed and stained as described in *METHODS* section. Populations of cells were selected by characteristic forward and side scatter properties and specific antibody staining fluorescence intensity. Specific staining for CD3^+^ T cells, CD19^+^ B cells, NK cells, DC, ILC2 and ILC3 are shown. (PDF 135 kb)

## Abbreviations

AHR: Airway hyper-responsiveness
ANOVA: Analysis of variance
BALF: Bronchoalveolar lavage fluid
DC: Dendritic cells
IL: Interleukin
ILC3: NKp46^+^ group 3 innate lymphoid cells
mAb: Monoclonal antibodies
NK cells: Natural killer cells
ODE: Organic dust extract
OVA: Ovalbumin
OVA-ODE: OVA-treated (asthmatic) mice
Th: T helper
TNF: Tumor necrosis factor
