# Supplementary figures and images for: Ovalbumin-sensitized mice have altered airway inflammation to agriculture organic dust
Source: Respir Res. 2019 Mar 7;20:51. doi: 10.1186/s12931-019-1015-0 (PMC6407255; doi:10.1186/s12931-019-1015-0)

Supplemental Figure 1.

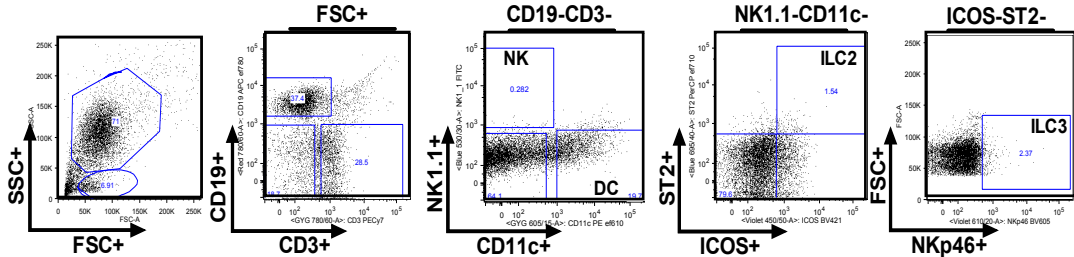

Supplement: Supplementary file 1 — Figure S1. A representative dot blot of gating strategy. Isolated lung cells were processed and stained as described in METHODS section. Populations of cells were selected by characteristic forward and side scatter properties and specific antibody staining fluorescence intensity. Specific staining for CD3+ T cells, CD19+ B cells, NK cells, DC, ILC2 and ILC3 are shown. (PDF 135 kb) [file 12931_2019_1015_MOESM1_ESM.pdf]
